# Sewage sludge pretreatment: current status and future prospects

**DOI:** 10.1007/s11356-023-28613-7

**Published:** 2023-07-15

**Authors:** Magdalena Ćwiertniewicz-Wojciechowska, Grzegorz Cema, Aleksandra Ziembińska-Buczyńska

**Affiliations:** Department of Environmental Biotechnology, Silesian University of Technology, Akademicka 2A, 44-100 Gliwice, Poland

**Keywords:** Anaerobic digestion, Methane fermentation, Hydrolysis, Biogas production, Wastewater, sCOD, Volatile fatty acids, Methane yield

## Abstract

Sewage sludge is regarded by wastewater treatment plants as problematic, from a financial and managerial point of view. Thus, a variety of disposal routes are used, but the most popular is methane fermentation. The proportion of macromolecular compounds in sewage sludges varies, and substrates treated in methane fermentation provide different amounts of biogas with various quality and quantity. Depending on the equipment and financial capabilities for methane fermentation, different methods of sewage sludge pretreatment are available. This review presents the challenges associated with the recalcitrant structure of sewage sludge and the presence of process inhibitors. We also examined the diverse methods of sewage sludge pretreatment that increase methane yield. Moreover, in the field of biological sewage sludge treatment, three future study propositions are proposed: improved pretreatment of sewage sludge using biological methods, assess the changes in microbial consortia caused with pretreatment methods, and verification of microbial impact on biomass degradation.

## Introduction

Continuously expanded exploitation of fossil fuels is leading to their depletion; hence, exploration into novel sources and methods for the provision of infinite electrical energy and heat inflow, while maintaining comparative usage to conventional sources, is of vital importance. Moreover, the exploitation is not only leading to depletion but also impacts the environment during the sourcing and use. Non-renewable fossil fuels affect climate changes by releasing large amounts of carbon dioxide into the air when are burned. That also forces us to minimize its usage and search for alternative solutions.

**Fig. 1:**
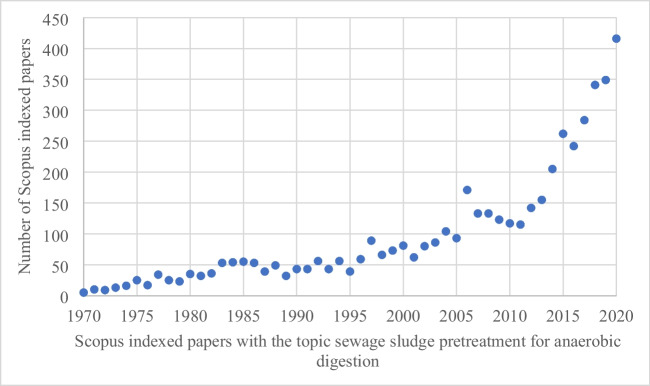
Numbers of research papers dealing with sewage sludge pretreatment for anaerobic digestion published between 1970 and 2022, according to Scopus

In general, human activity creates a vast amount of wastes, which gives rise to potential opportunities in the circular economy. The wastewaters are produced daily in domestic households, industries, and entertainment sectors, to name a few, which are treated in a wastewater treatment plant. However, a common side product of such treatment is the generation of sewage sludge that has the potential to be useful in biogas production. In recent years, biogas production by anaerobic digestion and its further usage is ever expanding and delving deeper in to unique and interesting areas of science. Figure 1 shows the number of papers published using the following keywords: sewage sludges, pretreatment, and anaerobic digestion.

Methane formation is an anaerobic microbial process that decreases the amount of dry and organic matter of substrates, causing stabilization of the properties of the sewage sludge. During this process, biogas is released which contains mainly methane and carbon dioxide at levels of 50–70% and 30–50% (*v*/*v*), respectively; however, residuals of hydrogen, hydrogen sulfide, and volatile organics also reside (Shrestha et al. 2020; Czatzkowska et al. 2020). The amount and quality of biogas are determined by the substrate characteristics and the ability of the fermenting microorganisms to conduct the following four phases: hydrolysis, acidogenesis, acetogenesis, and methanogenesis. According to reports, methane production during anaerobic digestion is mostly dependent on the composition of the sewage sludge used in the fermentation process and methods used to treat the biomass (Li et al. 2019; Park et al. 2021; Liu et al. 2021b; Akbay et al. 2021). A high amount of organic matter influences the efficacy and rate of the anaerobic digestion, thus sludge treatment. Moreover, usually, the sludges produced by the wastewater treatment plants contain considerable quantities of heavy metals, pathogens, and bacteria (Liew et al. 2022). Generally, recalcitrant and low degradable biomass limits the hydrolysis stage, which impacts anaerobic digestion. During this stage, cell walls are destroyed and extracellular polymeric substances (EPSs) are released and degraded by acidogenic microorganisms, which employ EPSs as organic material. This is an important mechanism due to compounds in sewage sludge being considered relatively unfavorable substrates for microbial degradation. This stems from their structure, which protects the cell from osmotic lysis (Appels et al. 2010). EPSs are known as the dewaterability disruptor of sludge that bind with bacterial cell walls or remain in suspension. They prevent desiccation of the bacterial cell and promote water binding, making sludges difficult to dewater. In the case of thermal methods, bonded and intracellular water is more easily released compared to other methods, i.e., chemical method (Pilli et al. 2014). Additionally, depending on the sewage sludge composition and limitations of microorganism consortia, it is difficult to convert all raw materials during hydrolysis and avoid organic matter in post-fermented sludges. Research has shown that hydrolysis is a slow and limiting phase of anaerobic digestion (Li et al. 2019; Junior et al. 2021); therefore, it is important to study over improvement of pretreatment methods or hydrolysis before anaerobic digestion. The utilization of biological, chemical, thermal, or mechanical methods for the disintegration of sludge flocs and the release of simpler compounds increase biogas production (Lindmark et al. 2012; Tsapekos et al. 2015; Shrestha et al. 2017; Liang et al. 2021). Nevertheless, anaerobic digestion is a popular treatment process due to proven efficiency, reduction of pollutants, stabilization of sludges, and reduction of sludges demanding utilization (Pilli et al. 2014; Patinvoh et al. 2016). Thus, it is important to develop biomass pretreatment that will allow more effective usage of biomass and increase biogas production using an eco-friendly method and low financial outlay. The first phase of fermentation determines the effectiveness of the whole process, where the decomposition of macromolecular compounds into monomers and smaller molecules used by microorganisms in the following stages (Ziemiński and Frąc, 2021; Neumann et al. 2016). In 1978, Haug et al. conducted the first pretreatment of biomass via thermal methodology for sludge dewaterability and biodegradability under anaerobic conditions. Since this development, numerous publications on this subject have been published, which propose a plethora of combined methods and the utilization of biomass sources. However, even though pretreatment research focusing on hydrolysis improvement has been conducted for the last 40 years, comprehensive verification and testing has yet to be achieved. Nowadays, the majority of studies examine various biomass and comparison of methods in order to enhance methane production, as well as degradation or transformation of hard to decompose compounds. Moreover, pretreatment methods must consider ecological and economic aspects, including the removal of heavy metals and pharmaceuticals. In addition, there are increasing opportunities to recover various useful substances such as volatile fatty acids (VFAs), nutrients (nitrogen, phosphorus, potassium) and enzymes (Ye et al. 2020; Sichler et al. 2021). This review examines sewage sludge pretreatment methods for methane fermentation with view on substances that present limitations on the process, i.e., cellulose, hemicellulose, proteins, and lipids. Furthermore, the advantages and disadvantages of sludge pretreatment are reviewed and their influence on the methane production yield.

## Methane fermentation of sewage sludge

The instantly increasing human population has caused an increase in wastewater and sewage sludge volumes generated post treatment (Duan et al. 2011). In 2010, the world’s population was 6.956.823.603, and in 2020, it increased to 7.794.798.739. On November 2022, the population reached 8 billion. According to Population Division of the Department of Economic and Social Affairs United Nations, the number of people will increase over the next few years, which will influence heavily the production of wastes and sewage sludge. According to World Population Prospects (2022), the growth to 8.5 billion in 2030 and 9.7 billion is expected by 2050.

In the last decade, the volumes of sewage sludge production generated in wastewater treatment plants (WWTPs) were increasing continuously, which is caused by the increasing population. Table 1 shows sludge production in chosen European countries between 2010 and 2019 (Eurostat, the statistical office of the European Union). That is why biogas production through anaerobic digestion becomes more interesting.

**Table 1:** Total sludge production in Europe (thousand tones), years 2010–2019 (Eurostat, the statistical office of the European Union, 2022)

Country	2010	2011	2012	2013	2014	2015	2016	2017	2018	2019
Bulgaria	49.80	51.40	59.30	60.30	54.90	57.40	65.80	68.60	53.10	n.a.
Czechia	196.30	217.90	263.30	260.10	238.59	210.24	206.71	223.27	228.22	221.09
Ireland	89.99	85.65	72.43	64.55	53.54	58.39	56.02	58.77	55.23	58.63
Germany	1 893.64	1 946.29	1 848.85	1 808.72	1 830.82	1 820.57	1 794.36	1,785.55	1 761.62	1 749.86
Estonia	18.80	18.30	21.70	17.00	19.91	19.11	18.65	20.94	25.54	24.94
Spain	1 355.10	1 331.60	1 233.40	1 122.60	1 131.60	1 152.60	1 174.40	1 192.00	1 210.40	n.a.
Lithuania	21.39	19.76	20.11	22.82	22.08	21.92	25.92	24.94	24.59	24.18
Hungary	170.34	168.33	160.60	170.47	163.12	177.70	217.96	266.84	233.66	227.89
Austria	262.80	n.a.	266.30	n.a.	239.04	n.a.	237.94	n.a.	234.48	233.56
Poland	526.70	519.20	533.30	540.30	556.00	568.00	568.33	584.45	583.07	574.64
Rumania	82.10	114.10	85.40	172.80	192.33	210.45	240.41	283.34	247.76	230.59
Slovakia	54.76	58.72	58.71	57.43	56.88	56.24	53.05	54.52	55.93	54.83
Finland	142.70	140.90	141.20	95.20	115.70	146.00	146.99	161.19	146.62	n.a.

*n.a.* data not available

### Factors impacting on the sewage sludge management

In biogas production, various sources of substrates are used depending on availability, geography, optional price and demand, type of active industry in the area, and possibilities for usage. These factors impact methane yield in relation to microorganisms’ potential such as the ability to grow in extreme conditions, incubation temperature, and environmental preferences (Passos et al. 2015). In methane fermentation, easily fermented substrates include livestock manure, food-processing wastes, and sewage sludge (Patinvoh et al. 2016; Córdoba et al. 2018).

Sewage sludges are composed of organic macromolecule compounds, varying in concentration and proportion. Wastewater flowing into WWTP is not only from private households but also from breweries, dairy factories, and paper industries, to name a few (Aski et al. 2020), which dictate the high concentration of lipids, proteins, cellulose and hemicellulose, and other polysaccharides in wastewater and then in sewage sludge. All sewage sludges are produced during wastewater treatment; hence, the composition of the sewage sludge is directly related to the composition of the wastewater that goes directly into the wastewater treatment plant. Thus, it can contain a qualitative and quantitative variety of lipids, pathogens, pharmaceuticals and their derivatives, or heavy metals. Methane fermentation of sewage sludge, other than its positive aspect for sludge stabilization and biogas production, creates wastes. These wastes depend on the degree of fermentation, calorific value, and element content such as phosphorus, potassium, calcium, and heavy metals, which can be managed, burned, or used as fertilizer. Moreover, wastewater treatment plants must contend with the presence of challenging compounds, such as insoluble proteins (about 30%, *w*/*w* TS in municipal primary sludge) (e.g., keratin) and polysaccharides (about 26%, *w*/*w* TS in municipal primary sludge) (e.g., cellulose) (Glińska et al. 2020). Recalcitrant form and difficulties in degradation of these compounds hinder the large amounts of dry matter and organic matter present in the post-fermented sewage sludge. As with almost every resource, sewage sludges must be pretreated to become efficient and safe biomass during and after the process of fermentation. These treatments must be cost-efficient and effective for various types of sewage sludge. Three groups of methods of sludge pretreatment are used, i.e., chemical, physical (including thermal and mechanical), and biological methods. Each provides different advantages and disadvantages in sewage sludge treatment, presented in Table 2. The applied impact is related to the amount of biogas produced and the level of stability of the sewage sludge. According to the literature, the most effective are thermal methods, which provide improvement in methane yield production up to 1000% (Appels et al. 2008; Nguyen et al. 2021), although the costs of energy needed to heat the sludges need to be considered. Mechanical methods provide usually not less than 100% improvement in methane production, and their use in the industry is most common (Yue et al. 2021). Usage of the chemical methods provides around 50% of the better methane production, but they are regarded to be harmful for the environment and sewage sludge itself (Zhang et al. 2023). Biological methods are not as effective as other methods mentioned above; however, they are regarded to be the safest and can be improved with other methods at a relatively low cost from other methods (Wandera et al. 2019; Liang et al. 2021). As mentioned previously, wastewater treatment plants choose pretreatment methods depending on their financial and technological possibilities, but also according to the sludge composition, mainly the presence of hard-to-decompose substances. The main purpose of the anaerobic digestion is to decrease the amount of organic substances present, i.e., in the sewage sludges, so their products could be used further in the future. Without the proper treatment, the large amount of organic matter residues remains, and eventual deposition in the environment may be dangerous for the soil and water. Sewage sludges that are not treated properly can cause odors, environmental pollution due to the recalcitrant leakage, and finally potentially hazardous influence on the human and animal health (Nguyen et al. 2021). Proper pretreatment provides not only the possibility for decreasing sludges volume directed from WWTPs but also their further usage due to stabilization of the sewage sludge properties during the anaerobic digestion, and the production of the biogas is its beneficial feature. The usage of the sewage sludges as a source for the methane production and providing its better composition contributes in a circular economy strategy.

**Table 2:** Comparison of biomass pretreatment methods described in the “Methods of sewage sludge treatment” section

Method	Used tools	Advantages	Disadvantages/challenges	Effectiveness	References
Chemical methods	HCl, H_2_SO_4_, H_3_PO_4_, HNO_3_, HNO_2_	-Hemicellulose degradation, improvement of proteinaceous biomass treatment	-The expensive process of acids recovery, destroying of devices (corrosion), occurrence of inhibitors	~50%	Dai et al. (2018), Taherzadeh and Jeihanipour (2012), Solarte-Toro et al. (2019), Zhang et al. (2023)
	NaOH, KOH, Ca(OH)_2_, Mg(OH)_2_, CaO, and ammonia	-Degradation of the lignin structure, internal surface area increase	-High costs of bases, inhibition of the methanogenesis using inappropriate concentration, occurrence of inhibitors		
Physical methods	Mechanical: ultrasonic, microwaves, milling, beating	-Increase of surface area, decrease of biomass size, decrease of biomass recalcitrance, possibility of using heat and electrical energy from biogas production, disruption of chemical bonds in the cell wall and membrane, increase of solubilization of sludge, increase of VS degradation, increase of methane production, reduction of retention time	-High investment and operating costs, consumption of enormous electric energy	>100%	Zawieja et al. (2019); Özön and Erdinçler (2019); Liu et al. (2020); Yue et al. (2021); Kazimierowicz et al. (2022)
	Thermal hydrolysis: steam explosion, 60–270°C, pressure 6–25 bar	-Partial solubilization and disintegration of biological cells, partial removal of micropollutants, high breakdown of the cell structure of sludge, release of intracellular bound water	-Odor generation, high operating costs, occurrence of inhibitors (i.e., ammonia)	Up to 1000%	Myszograj and Płuciennik-Koropczuk (2023); Kakar et al. (2022); Gahlot et al. (2022); Taboada-Santos et al. (2019); Nguyen et al. (2021)
Biological methods	Bacteria, fungi, enzymes	-Degradation of lignocellulosic biomass (hemicellulose, cellulose, and lignin), reduction of inhibitors	-A long time of culturing and high cultivating demands, long treatment time, difficulties with process modelling, high costs of enzymes, low solubilization yield	~10%	Agabo-Garciá et al. (2019); dos Santos Ferreira et al. (2020); Kumar and Gopal (2015); Jiang et al. (2022)

#### Lignocellulose complex

Cellulose is a troublesome compound, which is a part of a lignocellulose complex that also contains hemicellulose and lignin. The combinative effect of these compounds negatively influences the effectiveness of lignocellulose decomposition. Lignin is regarded as the most recalcitrant compound to undergo biochemical decomposition (Wagland et al. 2011; Li et al. 2021). Lignocellulose contains ca. 40–60% cellulose and 20–40% hemicellulose; hence, it is a potential carbon source for biogas production (Kang et al. 2014). According to Zhen et al. (2017), the utilization of lignocellulose as a substrate promotes the production of biogas containing approx. 50–70% CH_4_ and 25–50% CO_2_. Lignocellulose degradation process is mainly associated with bacteria and fungi performance, and isolation of enzymes produced by microorganisms (Zhang et al. 2018). Although lignocellulose is a limiting compound, thus for proper usage, it is necessary to convert it into simpler and more soluble compounds, which requires lignin removal methodology (Shah et al. 2018). Wagland et al. (2011) showed that high temperature is the most efficient way for the decomposition of such biomass. In the case of other approaches, including chemical methods, pretreatment with high energy may assist the degradation process. An alternative for high-temperature pretreatment or assisted temperature pretreatment is the chemical method, where incorporation of acids and bases removes or degrades various compounds. For lignin removal or pretreatment from sewage sludges, the alkaline pretreatment approach is preferred, which mainly utilizes KOH, Ca(OH)_2_, and NaOH (Dai et al. 2018). Ethanol, benzene, and ethylene glycol have also been investigated for lignin removal (Taherzadeh and Karimi, 2008). The possibility of biomass fermentation increases in the presence of bases due to lignin degradation (Patinvoh et al. 2016). However, alkaline treatment decreases the degree of polymerization of lignocellulosic biomass compounds, promoting lignin removal, and increases organic matter solubility in sludge and easier cellulose degradation by enzymatic and microbial impact (Liu et al. 2021a). Thus, alkaline treatment method is a promising and efficient way to lignocellulosic biomass decomposition. In the reference to the chemical treatment, the utilization of acids in pretreatment and assisted pretreatment methodology increases the fragility of the biomass in fermentation and promotes intramolecular bond cleavage between lignin, hemicellulose, and cellulose in the cell wall (Taherzadeh and Jeihanipour 2012). The disturbance over chemical construction enables the more efficient operation of cellulolytic enzymes. It is known that the presence of acids contributes to the hydrolysis of celluloses and hemicelluloses but does not dissolve lignin, only contributing to its partial destruction (Solarte-Toro et al. 2019). Fernandes et al. (2012) reported that biomass adapted to 4.9 g NH_4_+-N*/L and a lack of inhibition of the hydrolysis of cellulose after the addition of ammonia concentration 2.4–7.8 g NH_4_+-N*/L. Free ammonia addition is known as an effective method to improve anaerobic methane production. Using it as a pretreatment method can enhance the fermentation process but also adapt biomass to handle any ammonia concentration (Wei et al. 2017a).

#### Proteins and lipids

Usually, the main part of the sewage sludge organic matter consists of carbohydrates; hence, numerous studies focus on the hydrolysis and decomposition of such compounds. However, in the case of proteinaceous sewage sludge, the amount of protein content exceeds that of carbohydrates. Proteinaceous sewage sludge originates from the dairy industry, and it is characterized as a valuable source for methane production, due to its high VFA content. In some industries, protein content in dairy wastewater is over 40% of total oxygen demand, but the addition of carbohydrate matter can increase protein conversion in the sewage sludge. Moreover, pH influences methane production through the composition of VFA (Liu et al. 2021a). The use of sludges rich in protein sources, such as keratin wastes (e.g., feathers), contains over 90% of crude protein instead, or mixed with sewage sludge may provide higher biogas production. Due to the insolubility of the crude protein, it is necessary to provide access for microorganisms, which allow effective decomposition and conversion into soluble oligomers (Patinvoh et al. 2016). According to Ma et al. (2019), alkaline fermentation leads to efficient VFA enhancement production providing a carbon source for biological nutrient removal and increased degradation of difficult to biodegrade organic matter. Reports have shown that increased VFA concentration contributes to intensification of biogas production (Worwag and Kwarciak-Kozłowska 2019).

The prevalence of proteins in sewage sludge can also inhibit the process. Inhibition of bacterial activity can occur when substrates and products are in significant concentrations. Thus, the properly chosen pretreatment method may be beneficial for the process, from the point of troubleshooting and economy of the treatment. Under such conditions, more VFA is produced, which may lead to inhibition of the process (Shah et al. 2018). When VFA concentration is too high (>3.6 g*/L (Zhang et al. 2001)), inhibition of methanogenesis and hydrolysis/acidogenesis occurs due to organic overloading (Fotidis et al. 2012). Methanogens are not able to remove hydrogen and volatile organic acids faster than they are produced, resulting in this compound accumulation that causes changes in pH and disturbance of the hydrolysis and acidogenesis stages (Ponsá et al. 2008). High concentrations of ammonia can also inhibit methanogenesis, which is commonly present in complex wastes, mainly in animal manures, black water, or waste oil from gastronomy (Belmonte et al. 2011). Shi et al. (2017) observed that the presence of free ammonia (0.045 g*/L) caused an inhibitory effect of methanogenesis and accumulation of VFAs. Belmonte et al. (2011) showed that the concentration of free ammonia above 0.04 g NH_3_-N*/L caused IC_50_ (concentration at which a substance exerts half of its maximal inhibitory effect) of 56–84% for methanogenic bacteria present in raw swine wastewater and 84–94% for treated swine wastewater.

## Methods of sewage sludge treatment

The pretreatment methods of sewage sludges are used to support the process of methane fermentation (Patinvoh et al. 2016; Wluka et al. 2021; Nguyen et al. 2021; Machnicka and Grübel 2023). Methods used for the pretreatment of biomass for biogas production on a large scale are chemical, or physical, including thermal and mechanical. Recently, more attention gains also biological methods, based on microorganisms possessing particular features and enzymes produced by them (Kumar and Gopal 2015; Junior et al. 2021; Nguyen et al. 2021). For now, biological methods find their appliance only in the lab scale. The effectiveness of these methods can be compared only with the impact on the precise substrate (Lee et al. 2010). The potential advantages and disadvantages of the proposed methods are compared in Table 2.

All described methods demand high investment or operating costs, but in the long-term appliance, advantages may be overall the negative aspects. There is no universal pretreatment method for all biomass types; thus, the methods described in Table 2 and the potential of their usage are usually different depending on the composition of biomass sources. Nevertheless, each of the presented provides an increase in methane production and improved sewage sludge stabilizations, which decrease WWTP overall costs.

### Chemical methods

Pretreatment using chemical methods commonly employs acids and bases, which decrease recalcitration, and improves decomposition or degradation of biomass. Depending on the composition and origin of the biomass, various solutions of different concentrations are used to increase decomposed solid compounds and methane yield. Unoptimized and unbalanced pretreatment methods can lead to the production of inhibitors, such as furfurals during high thermal acidic pretreatment of the lignocellulosic substrate in order to degrade cellulose and hemicellulose (Chen et al. 2007), or VFA during alkali pretreatment of proteinaceous sludge. From a financial point of view, chemical methods are highly desirable. They are easily modified and result in high methane yield. In most reported cases, chemical pretreatment avoids subjection to thermal conditions. The most commonly used chemicals are HCl, H_2_SO_4_, H_3_PO_4_, HNO_3_, and HNO_2_ for acid pretreatment and NaOH, KOH, Ca(OH)_2_, Mg(OH)_2_, CaO, and ammonia for alkali pretreatment. Lü et al. (2007) examined alkali pretreatment and determined that using ammonia in protein hydrolysis caused its biodegradation. In the case of lipids, different concentrations of ammonia displayed varying degrees of influence on easy and hard biodegradable lipids. Wei et al. (2017a) verified the influence of free ammonia on methane production, where the addition of 250 mg NH_3_-N/L of the free ammonia was effective in enhancing methane fermentation. The greatest improvement of biochemical methane potential and hydrolysis rate was determined for 420–680 mg NH_3_-N/L.

Shah et al. (2018) studied alkali reagents of various concentrations in conjunction with different heating processes, including water bath, autoclaving and short time microwaving. The results showed that the highest delignification, compared to the other methods, was obtained using 2% NaOH and short time microwave heating process, providing biogas yield of 560 mL/g VS.

Wei et al. (2017b) showed that during acid pretreatment, addition of N/L free nitrous acid increased methane production by 16±1% and 16–17% reduction of dewatered sludge for final disposal. According to Zahedi et al. (2016), addition of free nitrous acid (2.49 mg N-HNO_2_/L) and incubation for 5 h increased the solubility of organic matter and methane production up to 25%.

Liu et al. (2021b) reported that pH regulation and chemical pretreatment were achieved using NaCl and NaOH. They determined that at pH = 11.0 the concentration of soluble proteins and VFA was higher than that at the pH = 3.0. Hence, pH modification impacted not only on VFA production but also the percentage of individual VFA. All acids and bases described above are used for sewage sludge treatment in research and industry. Dai et al. (2018) researched chemical and biological pretreatment of rice straw. Pretreatment using cellulase with NaOH, HCl, and CO(NH_2_) increased biogas production under anaerobic conditions. HCl dissolved hemicellulose by approx. 12.5–7.1%. After cellulase pretreatment, cellulose degradation was 38.3–10.9%. Pretreatment using 6% NaOH resulted in TS and VS removal of 53.8% and 36.8%, respectively. Song et al. (2014) compared seven chemicals alkali and acid pretreatment methods of various concentrations: H_2_SO_4_, HCl, H_2_O_2_, and CH_3_COOH at concentrations of 1%, 2%, 3%, and 4% (*w*/*w*) for acid pretreatment and NaOH, Ca(OH)_2_, and NH_3_*H_2_O at concentrations of 4%, 6%, 8%, and 10% (*w*/*w*) for alkaline pretreatment. The results showed that H_2_O_2_ and Ca(OH)_2_ were the most efficient, giving 216.7 and 206.6 mL of CH_4_/g VS.

### Physical methods

The complex structure of sewage sludge compounds prevents effective processing, forcing mechanical, ultrasound, microwave, or explosion treatment before anaerobic digestion. The main goal for pretreatment of sewage sludge is to increase the accessibility of the surface area and pore size, and cell walls break down, allowing microorganisms access to nutrients, thus improving biomass decomposition (Zhen et al. 2017). Physical pretreatment methods are preferable to treat difficult biomass, such as hard food wastes; agricultural residues from straw, crops, and wood; and forest residues (Rodriguez et al. 2017). Mechanical methods of disintegration reduce particle size due to external stress and pressure, converting compact masses of biomass into more soluble material (Shrestha et al. 2020). The size of particles impacts biogas production, where larger particles result in lower chemical oxygen demand (Nguyen et al. 2021). Ultrasound is considered an effective mechanical method. During ultrasonication, microbubbles that form collapse after reaching the critical size and cavitation occur. This process increased with increasing temperature and pressure, initiating hydro-mechanical shear forces and the presence of reactive radicals (H∙ and ∙OH). The decomposition of sludge flocs and release of intercellular material by physical methods have the hydro-mechanical shear forces and oxidizing influence of radicals, but the greatest impact has hydro-thermal shear forces (Pilli et al. 2011).

Another commonly used method of pretreatment involves microwaves, where the generated heat causes disintegration of compounds via hydrogen bond cleavage due to changes in dipole orientation at the polarized side chains in the cell membranes of macromolecules (Park et al. 2004, Serrano et al. 2016). Microwave is considered a promising pretreatment method and is normally used in crop straw pretreatment (Liu et al. 2021a). It is a rapid heating source, promoting thermal and nonthermal effects, resulting in enhanced biogas production, and it is regarded a supporting method for chemical treatment (Xu, 2015; Zaidi et al. 2019). Elalami et al. (2020) determined that using a single microwave or ultrasound pretreatment was less effective than the combined methods with alkali pretreatment. Moreover, mild microwave or ultrasonic pretreatment did not affect lignin degradation; however, the utilization of NaOH showed a substantial effect. Furthermore, alkali pretreatment reduces lipid content by saponification.

For mechanical pretreatment, various beaters or mills can be employed. Rodriguez et al. (2018) used the Hollander beater for anaerobic digestion of algae *Pelvetia canaliculata*. This resulted in maximum methane production of 283 mL/g VS and increased methane yield up to 43% in reference to non-pretreated algae. The Hollander beater was used also by Tedesco et al. (2014), where after pretreatment at 50°C for 10 min, biogas production increases by 52% and methane yield by 53% compared to the control sample. Rice straw biogas production using biological pretreatment supported by milling was performed by Mustafa et al. (2017). First biomass was treated with fungal strains and then prior into a mill. This combination increased methane production by 165%. It is known that particle size matters. Izumi et al. (2010) verified its effect on anaerobic digestion of food wastes. Bead milling was used and enhanced methane production by 28%, compared to the non-pretreated sample. Depending on the used biomass, physical method may give different results, so also comparison inside the group of methods is studied. Suresh et al. (2013) showed higher methane production using green algae *Ettlia* sp. as a substrate with the pretreatment under autoclave conditions compared to using sonicated residue and 250 W microwave. Montingelli et al. (2015) verified the influence of microwaves, ball milling, and beating on micro-algae biomass and determined that beating increased methane yield up to 37%, but ball milling and microwave method gave lower methane yield compared to biomass without pretreatment.

Among all pretreatment methods, thermal hydrolytic pretreatment reduces the VS and enhances biogas production (Chen et al. 2018). Thermal hydrolysis is a commonly used pretreatment method in industry for effective sludge treatment (Liang et al. 2021). Such pretreatment methodologies improve dewaterability, solubilization, and liquefaction of organic matter (Pilli et al. 2014). Additionally, thermal hydrolysis decreases particle size and viscosity, which increases access of the microbes and enzymes to the substrate (Chen et al. 2019). Reports have shown that thermal hydrolysis used on a full scale at WWTP increases the net electrical production of biogas by 20% (Tyagi and Lo 2013; Liang et al. 2021). The temperature range of thermal pretreatment is between 60 and 270°C, which categorizes pretreatment into low- (below 100°C) and high-temperature thermal pretreatment (above 100°C). High-temperature thermal pretreatment destroys sewage sludge particles and releases intracellular water, which is also achieved by particle collisions that causes gel structure destruction (Chu et al. 1999). Furthermore, an increase in temperature increases the chemical oxygen demand (Perez-Elvira et al. 2010). An increase in carbohydrate concentration was observed up to 130 °C and then decreases above 165°C. The results were obtained using spectrophotometric techniques by quantifying carbonyl groups (Pilli et al. 2014). The best results in sludge biodegradability, dewaterability, and biogas production (an increase of 40–80%) were obtained using pretreatment in 160 for 30 min and 180°C for 60 min. Low- and high-temperature pretreatment differs in the treatment time which is needed to obtain the same biogas generation. In the case of low-temperature pretreatment, the time period use can be hours or even days, with an increase of 20–50% in biogas production in comparison to samples never treated. The anaerobic pretreatment may be conducted in mesophilic and thermophilic conditions, or of their combination as two-stage anaerobic digestion. Under these conditions, hyperthermophilic chamber is used, followed by a mesophilic digester, causing an increase of solid-state hydrolysis, acidogenesis in thermophilic conditions, and then methanogenesis in a mesophilic environment (Handous et al. 2019). Ruffino et al. (2020) compared temperature-phased anaerobic digestion (TPAD) regarding conventional anaerobic digestion and showed higher efficiency in volatile solid (VS) reduction and methane generation. Amodeo et al. (2021) studied two different organic fractions of municipal solid wastes in co-digestion with digested sludge and determined an increase of methane production for both substrates at 55°C of solid retention time. Xiao et al. (2018) compared TPAD and food wastes with single-stage digestion in mesophilic and thermophilic anaerobic digestion and determined that lower methane yields were obtained when temperature-phased pretreatment was used. All thermal pretreatment methods influence methane production, enhance VFA generation, solubilization, and soluble chemical oxygen demand (sCOD) (Pilli et al. 2014; Junior et al. 2021). Low-temperature pretreatment increases sCOD, proteins, and sludge solubilization, but at a very low level, compared to high-temperature treatment (Chen et al. 2018). Thermal pretreatment consists of heat energy provided by heat exchangers or steam explosion, generating high pressure and heat. Such pretreatment methods enhance solubilization. The highest methane yield was observed after pretreatment for 15 min at 10 bar and 10 min at 15 bar. Similarly, studies conducted by Lizasoain et al. (2016) confirmed a positive influence of steam explosion regarding reed biomass, in which increased methane yield up to 89% was obtained after 15 min pretreatment at 200°C. A comparative study of steam explosion at 170°C and regular thermal sludge treatment in the autoclave (70, 100, and 125°C) was conducted by Liu et al. (2019). It was found that steam explosion had the potential to pretreat granular aerobic biomass with low methane yield caused by high mineral content. According to reports, the method that considers mineral content may be useful for resource recovery.

### Biological methods

Over the past few years, biological method using microorganisms and enzymes under aerobic and anaerobic conditions is a promising biomass pretreatment method. During the process, extensive degradation of lignin conducted by white-rot fungi and their enzymes responsible for the decay was observed (Adney et al. 2009). Fungi are considered to be the most effective microorganisms in biodegradation of lignocellulosic biomass, but other microorganisms have also their part. The influence of microorganisms is highly effective and improves both the pretreatment and fermentation process. Under optimal conditions, microorganisms stop the activity of inhibitors if they are biodegradable, or accumulated it while the concentration of non-biodegradable inhibitors is low. A high concentration of inhibitors leads to microbe death (Chen et al. 2007). Microorganisms can originate from various environments such as soil, manure, compost, food wastes, and sewage sludges (Kumar and Gopal 2015; Pessuto et al. 2016; Rodriguez et al. 2017; Baba et al. 2017; Zhang et al. 2018). Since microorganisms are already present in the environment, their addition cannot lead to microbial pollution, but the improper number, causing disturbance in the community, may decrease process efficiency. Similarly, as with chemical and physical pretreatment, biological methods may provide positive or negative biogas production effects. Microbial consortia during biogas production undergo various processes, which depend on the accessibility of nutrients and proper process conditions. Any disturbance in the microbial balance or presence of inhibitors may stop the biogas production process, or change its composition. As mentioned, microorganisms can take part in substrate treatment under aerobic or anaerobic conditions. Aerobic pretreatment consists of oxygenation, through the injection of oxygen into the pre-fermentation reactor, which improves the hydrolysis of macromolecular organic compounds by increasing the autochthonous microorganisms. Oxygenation, also called micro-aeration pretreatment, not only impacts microbial activity and diversity, causing hydrolysis increase, but also methane production (Nguyen and Khanal 2018). Micro-aeration stabilizes the liberation of exoenzymes, which biodegrade compounds that remain recalcitrant under anaerobic conditions (Ahn et al. 2014). Supporting impact on organic compound treatment in the aerobic pretreatment has a high temperature, which stimulates microbial consortia to produce enzymes (i.e., proteases) responsible for improving biomass solubility and organic compound degradation (Neumann et al. 2016). Nguyen et al. (2019) suggested that such pretreatment method could be used as an effective control strategy when the organic loading rate was high, thus, confirming rapid VFA conversion, followed by methane production, conducted by facultative anaerobic microorganisms and hydrogenotrophic methanogens. Using limited micro-aeration in to corn straw anaerobic digestion produces the following maximum results: 216.8 mL/g VS of methane yield and 54.3% removal of VS (Fu et al. 2016). Fu et al. (2016) showed changes in the microbial community structure and improvement of specific methanogenic activity after micro-aeration. Under micro-aerobic conditions, increased numbers of phylum *Firmicutes*, class *Clostridia*, and order *Clostridiales* were observed (Mustapha et al. 2018). As mentioned, micro-aeration at high temperature generates positive results and high potential, but research (Table 3; Ding et al. 2017; Liang et al. 2021) confirms high methane yield when using only high temperature and microorganisms. Biological pretreatment can proceed by the aforementioned methods described, using inoculation of the biomass with microorganisms, their activity products (isolated enzymes), or commercially available enzymes. According to reports, the basic assumption is that microorganisms present in the substrate use available compounds as food, allowing the use of the biodegradable products by other microorganisms, which undergo the following stages. The addition of extra microorganisms can disturb the balance in the microbial community of sewage sludge but also improve the process. The influence of microorganism inoculation is noticeable when using autochthonous microorganisms according to Pessuto et al. (2016) for anaerobic digestion of swine manure, where an increase in biogas and methane production was determined.

**Table 3:** Effect of soluble oxygen demand and VFA concentration and methane yield depending on the pretreatment method

Methods type	Biomass	Methods of treatment	Time of treatment	Temperature	sCOD before pretreatment	sCOD after pretreatment	VFA before pretreatment	VFA after pretreatment	Biogas yield, % (calculated)	References
Chemical methods	Sewage sludge	0.2 g β-cyclodextrin/g VS	8 days	35 ± 2 °C	53.7 ± 6 mg/L	-	220 mg COD/L	4200 g/g VS/g β-cyclodextrin	β-cyclodextrin inhibited methanogens activity (methane production decreased by 27.9%)	Yang et al. (2012)
	Waste-activated sludge	pH= 10	10 min	60 °C	2071 mg/L	~14019 mg/L		-	Methane production increased by 43.61%	Tulun and Bilgin, (2019)
		pH= 5	15 min	40 °C		9403 mg/L			Methane production increased by 12.34%	
		pH= 10	7 days	27–34 °C	940 mg/L	13390 mg/L	60.2 mg COD/L	2145 mg COD/L	Methane production increased by 51.94%	Presti et al. (2021)
	Sewage sludge + seeding sludge	pH= 10	8 days	37 °C	124.7 ± 14.34 mg/L; 398 **±** 31.25 mg/L	6448 ± 72.37 mg/L	722.3 ± 34.21 mg COD/L	3762 ± 204.86 mg COD/L	Lack of production	Ma et al. (2019)
Physicial methods/mechanical methods	Waste-activated sludge	Ultrasonic denisty (1.0 kW/L) + pH= 10	72 h	20 ± 1 °C	-	-	1275 mg COD/L	3109.8 mg COD/L	-	Yan et al. (2010)
		Thermal pretreatment	1 h	170 °C	-	-	-	8340 mg COD/L	Biogas production increased by 67.8%	Qiao et al. (2011)
	Sewage sludge (1 g VS substrate/g VS inoculum)	Thermal pretreatment	15 min	120 °C	17020 ± 180 mg/L	19680 + 540 mg/L	1800–2200 mg COD/L	2200–2600 mg COD/L	-	Iglesias-Iglesias et al. (2019)
	Primary sludge	Steam explosion (10 bar)	15 min	280 °C	Increase of sCOD by 53%	-	-	-	Methane production increased by 224% (380 mL CH4/g VS)	Aski et al. (2020)
		Steam explosion (15 bar)	10min		Increase of sCOD by 57%				Methane production increased by 205% (358 mL CH4/g VS)	
	Waste-activated sludge	Steam explosion (10 bar)	15 min		Increase of sCOD by 52%	-			Methane production increased by 169% (315 mL CH4/g VS)	
		Steam explosion (15 bar)	10min		Increase of sCOD 54%				Methane production increased by 185% (334 mL CH4/g VS)	
	Sewage sludge	Ultrasounds (27 kHz, 200 W/L)	2.5 min	20 °C	620 mg/L	2100 mg/L	-	-	Methane production increased by 151% (8.09 mL CH4/g sCOD)	Grönroos et al. (2005)
			10 min	60 °C		4200 mg/L				
Physicial method/thermal method	Thickened sludge	Low-temperature thermal treatment	15 min	70 °C	400 mg/L	650 mg/L	75 mg COD/L	111 mg COD/L	Lack of significant increase of methane production	Appels et al. (2010)
			30			800 mg/L		618 mg COD/L		
			60			1150 mg/L		1071 mg COD/L		
			15	80 °C		1350 mg/L		988 mg COD/L		
			30			2050 mg/L		1177 mg COD/L	Methane production increased by 37% (48.02 mL/g ODS)	
			60			8200 mg/L		1277 mg COD/L	Methane production increased by 117% (75.64 mL/g ODS)	
			15	90 °C		1600 mg/L		1733 mg COD/L	Methane production increased by 120% (76.69 mL/g ODS)	
			30			7200 mg/L		1963 mg COD/L	Methane production increased by 307% (141.94 mL/g ODS)	
			60			10250 mg/L		2744 mg COD/L	Methane production increased by 984% (377.56 mL/g ODS)	
	Waste-activated sludge	Low-temperature thermal treatment + alkali pretreatment (NaOH/Ca(OH)_2_=1:4)	6	80 °C	765 mg/L	-	225.4 mg COD/L	~700 mg COD/L	Methane production increased by 129.1%	Zou et al. (2020)
		Low-temperature thermal treatment + alkali pretreatment (NaOH/Ca(OH)_2_=2:3)						~700 mg COD/L	Methane production increased by 146.1%	
		Low-temperature thermal treatment + alkali pretreatment (NaOH/Ca(OH)_2_=1:1)						~700 mg COD/L	Methane production increased by 154.6%	
		Low-temperature thermal treatment + alkali pretreatment (NaOH/Ca(OH)_2_=3:2)						~730 mg COD/L	Methane production increased by 163.8%	
		Low-temperature thermal treatment + alkali pretreatment (NaOH/Ca(OH)_2_=4:1)						~670 mg COD/L	Methane production increased by 171.7%	
		Low-temperature thermal treatment	30 min	60 °C	150 mg/L	3510 mg/L	-	-	Methane production increased by 31% (117.5 mL/g VS)	Zheng et al. (2021)
		High-temperature thermal treatment		160 °C		5700 mg/L		-	Methane production increased by 75% (156.4 mL/g VS)	
Biological methods	Raw sewage sludge	Microbial electrochemical system	6 days	35 + 2 °C	Increase of sCOD by 31%			-	Methane production increased by 47 and 56%	Vu and Min, (2019)
	Secondary sludge	Biological hydrolysis	6 days	35 °C	-	-	24.5 mg/L	666.5 mg/L	~95 mL/h	Chen and Chang, (2018)
				42 °C				918.4 mg/L	Biogas production increased by 5% with increase of the temp. (~100 mL/h)	
				55 °C				1398.5 mg/L	Biogas production increased by 10% with increase of the temp. (~ 105 mL/h)	
		Hydrolytic microorganisms + thermal treatment	6 days	35 °C	175.2 ± 38.2 mg/L	3314.5 ± 683.4 mg/L	100 mg/L	1021 ± 155.2 mg/L	Very low methane yield, decreasing with increase of temperature	Ding et al. (2017)
				42 °C		2857 ± 193.5 mg/L		1362.5 ± 165.2 mg/L		
				55 °C		4518.5 ± 190.6 mg/L		1423 ± 117.4 mg/L		
	Thickened sludge	Hyperthermophilic hydrolysis + acetogenic microorganisms	5 days	70 °C -> 35 °C	7100 ± 1400 mg/L	43600 ± 17500 mg/L	1920 ± 400 mg COD/L	57000 ± 9000 mg COD/L	Methane production increased by 462% (152 mL CH4/g VS)	Mahdy et al. (2019)
		Two-stage process	48 h	50±1 °C	244.21 ± 12.65	7850.96 mg/L	190.85 ± 1.93 mg COD/L	5699.44 mg COD/L	-	Xiong et al. (2012)
		Hyperthermophilic hydrolysis + acetogenic microorganisms	5 days	70 °C -> 37 °C	8600 ± 2000 mg/L	12000 ± 2000 mg/L	-	2567 ± 440 mg COD/L	Methane production increased by 375% (176 mL/g VS)	Wandera et al. (2019)
	Waste-activated sludge	Thermal hydrolysis, fungal fermentation (*Aspergillus niger*)	3 h (1 h each temp.)	140 °C	-	-	-	-	Methane production increased by 7% (302.9 ± 19.9 mL biogas/g COD)	Liang et al. (2021)
				160 °C					Methane production increased by 13% (312.3 ± 21.2 mL biogas/g COD)	
				180 °C					Methane production increased by 5% (298.1 ± 11.0 mL biogas/g COD)	

*sCOD* soluble chemical oxygen demand, *VFA* volatile fatty acids

Supporting the pretreatment with enzymes has gained lot of interest. The addition of hydrolytic enzymes provides enhanced hydrolysis and increases biogas production, sludge solubilization, and degradation of the extracellular polymeric substances (EPSs) (Yin et al. 2016). Enzymes may be an alternative for thermochemical methods of hydrolysis, diminishing overall costs, as the enzymes may be produced by a wide range of bacteria and fungi (Miao et al. 2013; Mahdy et al. 2014).

There are 4 ways of enzyme addition methods according to Brémond et al. (2018), which rely on (1) addition to a dedicated pretreatment vessel, (2) direct addition to the hydrolysis and acidification vessel over a two-stage process, (3) direct addition to the digester in a single-stage process, and (4) addition to the recirculated anaerobic digestion leachate. Depending on the substrate, the composition and parameters of enzymes used should be optimized, such as isoelectric point, specificity, activity, and temperature (Divya et al. 2015). The research conducted by Zhao et al. (2018) compared chemical and biological pretreatment using enzymes, fungi, and alkali (NaOH) and combined NaOH with both fungi and enzymes. The combination of the two enzymes gave methane yield of approx. 276.16 and 273.75 mL/g TS, respectively. The presence of NaOH gave the same result as the first obtained during enzyme pretreatment, confirming that chemical pretreatment could be replaced by a safer method. NaOH combined with one enzyme pretreatment method increased methane production to over 20.24% compared to the control, but fungal treatment decreased biogas production and inhibited the process. Enzymes were used also by Moon and Song (2011), where hydrolysis reaction was conducted for 10 h. A mixture of enzymes (carbohydrase, protease, and lipase in ratio 1:2:1) was examined, generating 0.35 L CH_4_/gCOD. Biological methods have already been studied, but compared to chemical or physical pretreatment, they seem to be equally popular.

Biological methods using enzymes are a promising approach, but their price is high, and the time of producing enzymes by microorganisms is long. Hence, the production and subsequent recovery of enzymes are reasonable from the economical point of view (Marín et al. 2018). Cellulases were extracted from the substrate, which was further tested for methane production. The authors obtained 552±66, 543±47, and 663±40 mL biogas/g VS for apple pomace, orange peel, and rice fiber after 25 days, respectively, confirming the positive influence of enzymes. The authors tested recovered enzymes, which determined their potential reusability. Table 3 shows examples of pretreatment methods, the concentration of VFAs, sCOD, and methane yield, depending on the used biomass and total and volatile solids.

Pretreatment methods are useful not only for increasing biomass fragility but also for heavy metals or pharmaceutical removal from the sludges to the liquid phase; such methods include steam explosion pretreatment. This approach employs high pressure and heat causing break down of sludge structures and contained compounds. The steam explosion eliminates the need for chemical usage; hence, it can be considered as an eco-friendly pretreatment method (Aski et al. 2020). Aski et al. (2020) confirmed that this method allowed for pharmaceutical and heavy metal removal, such as ibuprofen (in 65 and 69%), acetaminophen (in 66 and 70%), amoxicillin (in 66 and 70%), lead (in 78 and 70%), and cadmium (in 79%) removal from primary sludge and waste-activated sludge by facilitating the leaching them out into the liquid phase. Physical pretreatment methods can be applied to sewage sludge pretreatment, as well as reduction of dangerous impact of pharmaceuticals, antibiotics, and heavy metals. Zhang et al. (2019) described that an influencing factor that reduces antibiotic resistance genes (ARGs) was present in chicken manure, when supported with activated carbon. The authors noted that ARG removal rate was obtained 87–95% in a digester with microwave pretreatment supported with activated carbon and 34–58% in the digester with substrate only microwaved. In other studies, Bao et al. (2020) showed an increase of VFA and sCOD concentration after ultrasound-alkali pretreatment of waste-activated sludge and methane yield of approx. 97±1.85% in reference to the control reactor. The research was conducted using a combined process of microbial electrolysis cell and anaerobic digestion. The obtained results suggest that such pretreatment not only supports the degradation of organic compounds and increases biogas production but also supports VFA recovery that becomes precursors for the production of higher-value biofuel and biochemical generation.

Currently, the methane produced in anaerobic digestion is used by wastewater treatment plants for its purposes, to provide heat and energy for WWTPs’ infrastructure, or it is simply burned. Future perspective of using biogas as green energy source for larger group of consumers should be considered. Improved production of biogas may fulfil the energy demand with environmentally friendly replacement for fossil fuels.

## Conclusion

Sludges for fermentation may contain inhibitors, indigestible, and hard to degrade compounds, but their presence should not determine the low effectiveness of the process. An efficient process may be possible with proper supporting methods, providing effective treatment of substrates, with high energy benefits. One of the critical aspects is high methane potential of the substrate, which is determined only after all possible pretreatment methods are tested, because certain methods may not impact, increase, or decrease biogas and methane production. All described methods improve hydrolysis and methane yield, but the best effects are obtained using mechanical methods. Such methods are expensive and require high energy contribution, which, in the circular economy, can be returned in biogas production. Chemical methods may be less effective when used separately, can cause the production of inhibitors, and destroy equipment and large investment and exploitation costs. Biological methods produce comparative methane yields to other pretreatment methods but may not be yet considered as an alternative method for chemical or physical pretreatment on a large scale. Moreover, they are costly and cannot be easily transformed from a lab scale into the industry. All methods provide higher efficiency when they are used in combination with the other techniques, from a different category. As methane fermentation is a biological process, any change may cause microbial consortia balance disturbance; thus, it should be obligatory to verify the condition of the biomass activity before and after pretreatment, revealing the pretreatment effect. Both, the influence of pretreatment on microorganisms consortium present in the sewage sludges, and the influence of the microorganisms on the biodegradability of sludge and composition changes. Additionally, the analysis for determining hydrolysis effectiveness should be systematized, which will allow for proper comparison of possible methods. The greatest challenge for sustainable and efficient anaerobic digestion is possessing energy and economic balance, when considering ecological aspects. Moreover, very often efficient pretreatment methods are expensive when tested and used already in lab scale; therefore, they may not be attractive for industries. Potential influence on the pretreated substrate environment should be considered and the possibility of recovery compounds, substrates, or as circular economy attitude. For future studies, more attention should be given to the improvement of biological pretreatment methods without using chemical supported methods, as well as conducting additional recovery of used materials for a more eco-friendly approach.

Figures and tables of this word can be found in online version of the paper
